# Unveiling the HSF1 Interaction Network: Key Regulators of Its Function in Cancer

**DOI:** 10.3390/cancers16234030

**Published:** 2024-11-30

**Authors:** Snezhana A. Vladimirova, Nadezhda E. Kokoreva, Irina V. Guzhova, Bashar A. Alhasan, Boris A. Margulis, Alina D. Nikotina

**Affiliations:** Institute of Cytology of Russian Academy of Sciences, Tikhoretsky Ave. 4, 194064 St. Petersburg, Russia; snezhana.alexandrovna@mail.ru (S.A.V.); kokoreva.nadya00@gmail.com (N.E.K.); irina.guzh@gmail.com (I.V.G.); alhassan.bashar.1994@gmail.com (B.A.A.); margulis@incras.ru (B.A.M.)

**Keywords:** HSF1, protein–protein interactions, cancer, HSP90

## Abstract

Heat shock factor 1 (HSF1) plays a central role in orchestrating the heat shock response (HSR), leading to the activation of multiple heat shock proteins (HSPs) genes and approximately thousands of other genes involved in various cellular functions. In cancer cells, HSPs play a particular role in coping with the accumulation of damaged proteins resulting from dysregulated translation and post-translational processes. This proteotoxic stress is a hallmark of cancer cells and causes constitutive activation of HSR. Beyond its role in the HSR, HSF1 regulates diverse processes critical for tumor cells, including proliferation, cell death, and drug resistance. Emerging evidence also highlights HSF1’s involvement in remodeling the tumor immune microenvironment as well as in the maintenance of cancer stem cells. Consequently, HSF1 has emerged as an attractive therapeutic target, prompting the development of specific HSF1 inhibitors that have progressed to clinical trials. Importantly, HSF1 possesses a broad interactome, forming protein–protein interactions (PPIs) with components of signaling pathways, transcription factors, and chromatin regulators. Many of these interactors modulate HSF1’s activity and HSF1-dependent gene expression and are well-recognized targets for cancer therapy. This review summarizes the current knowledge on HSF1 interactions with molecular chaperones, protein kinases, and other regulatory proteins. Understanding the key HSF1 interactions promoting cancer progression, along with identifying factors that disrupt these protein complexes, may offer valuable insights for developing innovative therapeutic strategies against cancer.

## Simple Summary

Cancer cells are susceptible to various kinds of stressors. They are able to overcome stressful conditions leading to impaired cellular protein integrity, largely due to the activity of heat shock proteins, whose main regulator of the synthesis is heat shock factor 1 (HSF1). Participation of HSF1 in the maintenance of tumor growth is also associated with its ability to regulate diverse processes critical for tumor cells, including proliferation, cell death, and drug resistance by diverse mechanisms. This makes HSF1 a promising target for therapy. However, the search for molecules capable of inhibiting HSF1 activity is hampered by a number of complexities. One approach to targeting HSF1 may be to disrupt protein-protein interactions with its interacting molecules, many of which modulate HSF1 activity and HSF1-dependent gene expression. This review summarizes the known data on HSF1 interactors and considers them from the point of view of the possibility of disrupting HSF1 activity and, consequently, suppress tumor progression.

## 1. Introduction

The heat shock factors (HSFs) constitute a family of transcription factors, comprising several homologous members. Among them, HSF1 is activated by a wide range of stressors and functions as the primary regulator of the evolutionarily conserved heat shock response (HSR) program [1,2]. In addition to its role in stress responses, HSF1 can also activate the expression of non-HSP genes in tumor cells, thereby regulating cell cycle progression, promoting epithelial–mesenchymal transition (EMT), and maintaining the stability of cancer stem cell populations [2,3,4]. HSF1 activation is tightly controlled by a complex regulatory system, which includes molecular chaperones and various enzymes that modify the protein as it translocates to the nucleus. Under stress conditions, HSF1 translocates from the cytoplasm to the nucleus, though the nuclear localization of HSF1 is typical in epithelial cells under non-stress conditions [5].

The molecular structure of HSF1 is largely disordered, except for the evolutionarily conserved domains. Of which, the N-terminal DNA-binding domain (DBD) forms a winged helix-turn-helix structure that binds to inverted nGAAn pentameric repeats on the promoter regions of target genes known as heat shock elements (HSE). HSF1 also contains the oligomerization domain HR-A/B comprising leucine zippers essential for its trimerization, while the unstructured regulatory domain (RD) senses heat and triggers the HSR. HSF1 also has binding sites for the basal transcriptional complex and multiple sites for post-translational modifications. Additionally, the HR-C domain of HSF1 inhibits spontaneous trimerization by sequestering HR-A/B, while the C-terminal transactivation domain (TAD), which is enriched in hydrophobic and acidic amino acids, is highly disordered and crucial for the HSF1 transcriptional activity in response to diverse stress conditions [6,7].

The regulation of HSF1 activity is a complex process that involves multiple factors, such as protein–protein interactions, its abundance, multimeric state, phosphorylation, nuclear translocation, and DNA-binding activity [8,9]. In cancer cells, post-translational modifications are one of the major instruments to regulate HSF1 activity [8]. These modifications regulate its cellular localization, binding to DNA, interaction with other proteins, transactivation, attenuation, and degradation. The most significant modification of HSF1 seems to be phosphorylation, as it can either activate the factor or, conversely, inhibit it. Phosphorylation at S230, S419, Thr142, S320, and S326 residues promotes HSF1 activation, while phosphorylation on S303, S307, S363, and S121 leads to HSF1 suppression [10]. Among these sites, phosphorylation at serine 326 (S326) is the most significant for HSF1 activation, as it stimulates the HSR and correlates with poor prognosis in cancer patients [11]. Several kinases are capable of phosphorylating HSF1 at this site, including AKT, ERK1/2, CDK8, MEK, mTORC1, DYRK2, and p38 MAPK [11,12,13]. Phosphorylation at S303 or S307 suppresses HSF1 transcriptional activity, hence, protein kinases, including ERK, GSK3, CK2, AMPKα (at S303 only), and p38 MAPK, may act as negative regulators of HSF1 activity [11,14,15].

While little is known about the regulation of HSF1 at the transcriptional level, a 2018 study demonstrated that oncogenic Notch1 binds to the HSF1 gene promoter and induces its expression [16]. In addition, the study of Hoj et al. in 2020 reported that the tyrosine kinase ABL2 is another upstream regulator of HSF1 gene expression, and notably, the activation of this program is required for lung adenocarcinoma cells to form brain metastases [17].

## 2. HSF1 as a Key Player in the Molecular Mechanisms of Cancer Progression

Cancer cells are characterized by high expression levels of HSF1. This is due to its role in numerous cellular processes associated with tumorigenesis.

**Figure 1:**
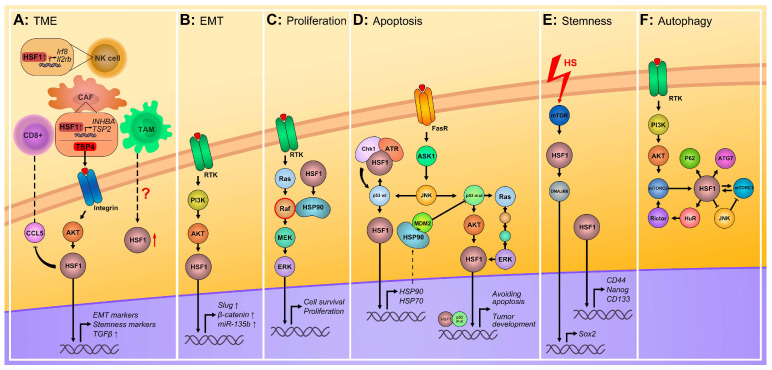
Molecular mechanisms cooperating with HSF1 in mammalian cells. HSF1 is involved in the regulation of interaction with TME by activating specific programs in both cancer and stroma cells (**A**); it enhances the migration and invasion ability of cancer cells by EMT program activation (**B**); it can promote higher proliferating activity by Ras/Raf/MEK/ERK signaling (**C**); participation of HSF1 in apoptotic pathways regulation depends on its coordination with p53 molecules (**D**); HSF1 can promote cancer cell stemness and upregulate stem factors (**E**); it regulates the activity of some participants in the autophagy process (**F**).

HSF1 supports cancer cells from different pathways (Figure 1). A growing body of evidence suggests that HSF1 is actively involved in the reprogramming of the tumor microenvironment (TME). First, it was found that cancer-associated fibroblasts (CAFs) exhibit increased HSF1 activity, which stimulates a program that promotes pro-tumorigenic stroma and tumor progression [18]. CAFs have also been shown to secrete thrombospondin 4 (TSP4), which activates AKT through integrin signaling. AKT, in turn, phosphorylates HSF1 at S326, resulting in elevated expression of EMT and stemness markers, as well as TGF-β [19].

In addition, HSF1 negatively regulates the expression of key mediators of the natural killers (NK) effector function, resulting in reduced cytokine production and cytotoxicity in HSF1-overexpressing NK cells [20]. Another way by which HSF1 helps tumor cells to evade immune surveillance is by suppressing the expression of CCL5, a CD8+ T-cell chemokine, thereby inhibiting the recruitment and infiltration of CD8+ T-cells into the tumor microenvironment [21]. Furthermore, we have recently demonstrated that co-cultivation of tumor cells with naive monocytes induces their conversion into tumor-associated macrophages (TAMs) and, consequently, promotes HSF1 activation in tumor cells, though the underlying mechanism remains to be elucidated [22,23] (Figure 1A).

Another critical HSF1-regulated process is EMT. For example, it was shown that Akt-dependent activation of HSF1 in HER2-amplified breast cancer cells promotes EMT via direct upregulation of the transcription factor Slug [24]. HSF1 can positively regulate β-catenin translation through increasing HuR expression in mammary epithelial and carcinoma cells [25] and can cause amplified expression of miR-135b that promotes hepatocellular carcinoma cell migration and invasion [26] (Figure 1B).

HSF1 is closely related to the regulation of the cell cycle and proliferation of tumor cells. HSF1 activation allows cancer cells to overcome cell cycle checkpoints and provides rapid proliferation by some mechanisms [27]. In ErbB2+ cells, for example, HSF1 upregulates HSP90, which forms a complex with Raf and stabilizes it. This event triggers the activation of Ras/Raf/MEK/ERK signaling and promotes the proliferation [28] (Figure 1C).

HSF1 has long been known to be involved in the regulation of apoptosis by controlling the expression of chaperones that suppress the pro-apoptotic factors and prevent the initiation of programmed cell death [29,30,31]. In this context, HSF1 has been reported to have physical interactions with both wild-type (wt) and mutant forms of p53, with these interplays playing a pivotal role in apoptosis regulation. For instance, following the activation of wt p53, it directly activates HSF1 that induces the expression of chaperones, including HSP90, which, in turn, interacts with MDM2 and inhibits its function as a p53 suppressor [32]. On the other hand, HSF1 forms a ternary complex with ATR and Chk1, which promotes p53 phosphorylation and activation [33]. Mutant p53 can also activate the MAPK and PI3K/AKT signaling pathways via EGFR/ERBB2, leading to HSF1 activation and HSPs expression, which contribute to tumor progression, chemoresistance, and apoptosis evasion [34] (Figure 1D).

Moreover, HSF1 is crucial for maintaining the cancer stem cell phenotype. Thus, the heat shock-independent activation of HSF1 augments the breast cancer stem cell phenotype and confers drug resistance [25,35]. It was demonstrated that HSF1 activation by mTOR (mammalian target of rapamycin) leads to higher expression of cancer stem-like cell markers by activating DNAJB8, which, in turn, enhances the expression of Sox2 in renal carcinoma cells under stressful conditions [36]. On the other hand, it was shown that HSF1’s impact on cell stemness depended on Hsp27, which has a crucial role in the resistance to apoptosis in CD133+ colon cancer cells and ALDH^high^ endometrioid adenocarcinoma cells [37] (Figure 1E).

Involvement of HSF1 in the autophagy process is based on its ability to regulate the activity of several participants, such as ATG7, p62, mTORC1 [38], and mTORC2 [39] (further details on HSF1 interaction with the mTOR complex are discussed in Section 3). Desai et al. showed that HSF1-dependent upregulation of *ATG7* gene expression is activated by carboplatin treatment, playing a cytoprotective role and conferring resistance to chemotherapy of breast cancer cells [40]. Another work demonstrated that the HSF1 stress response pathway mediates p62 phosphorylation and results in the autophagic clearance of harmful proteins, which underlines the diverse functions of HSF1 in proteostasis regulation [41] (Figure 1F).

## 3. Functional Interactors of HSF1 in Cancer Cells

Protein–protein interactions are the fundamental basis of signal transduction in various cellular processes [42]. Aberrations in this network contribute to the development of neurodegenerative disorders, infectious diseases, and cancer. The fact that malignant transformation is characterized by significant changes in the interactions within the cellular proteostasis systems was revealed by the group of Chiosis and colleagues’ study of a network of chaperone and co-chaperone interactions, termed the “chaperome”. The authors demonstrated that alterations in the cellular environmental conditions (e.g., MYC activation) can lead to the reorganization of this network and the formation of more stable complexes, termed the “epichaperome”. The epichaperome defines the relevance of the chaperone network for tumor cell survival, which outlines its diagnostic and therapeutic potential in cancer [43].

PPIs can be classified by such factors as the nature of the interacting protein partners (homo- or hetero-oligomers), the stability of the protein complexes (obligate or non-obligate), and their duration (permanent or transient) [44,45]. The HSF1 interactome is highly dynamic, consisting of a wide variety of PPIs that can vary significantly depending on environmental conditions [46]. In this section, we have described interactions involving HSF1 as part of the chaperone complex, as part of complexes assembled on the promoter region of target genes, as well as interactions with various kinases that phosphorylate HSF1. Brief characteristics of these interactions are summarized in Table 1.

### 3.1. Chaperones

**Figure 2:**
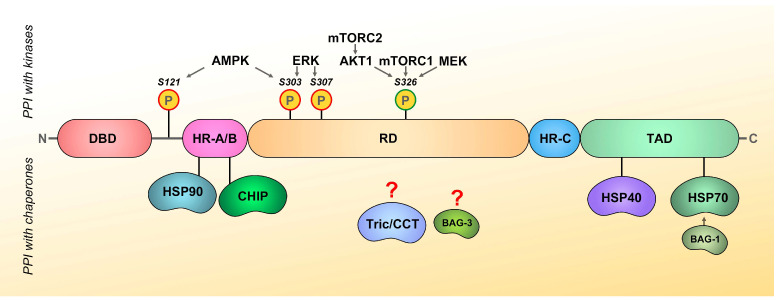
Schematic domain structure of HSF1 and its main PPI. The HSF1 molecule contains a DNA binding domain (DBD), oligomerization domains (HR-A/B and HR-C), a regulatory domain, and a trans-activating domain (TAD). The most significant modification sites of the molecule include S326, through which activating phosphorylation occurs by AKT1, MEK, and mTORC1, and S303, S307, and S121, whose phosphorylation by ERK and AMPK leads to factor inhibition. The chaperone binding sites are located at the HR-A/B (for HSP90 and CHIP) domain or at TAD (for HSP40, HSP70, and its co-chaperone BAG-1). The binding sites on HSF1 molecules for TRiC/CCT and BAG-3 are still not elucidated.

According to the chaperone titration model of heat shock response, HSF1 remains in monomeric/inactive cytoplasmic form through intramolecular interactions between HR-A/B and HR-C domains, and also through associating with chaperones (HSP90, HSP70, HSP40) or chaperonins (TRiC/CCT) that can negatively regulate the factor trimerization and activation [8,47] (Figure 2). This creates a feedback loop to rapidly induce the heat shock response under stressful conditions. Exposure to a wide variety of stresses disrupts cellular proteostasis and causes the recruitment of chaperones to misfolded proteins, which in turn leads to the release and activation of HSF1 in a posttranslational manner [48].

**HSP90** is an essential chaperone that ensures proper folding of its client proteins, many of which are key components of signaling pathways that contribute to oncogenesis. In unstressed cells, HSP90 forms a complex with HSF1, acting either independently or in conjunction with multichaperone complexes as a primary repressor of HSF1 [49]. Upon exposure to stress, HSP90 dissociates from HSF1, which represents a critical step in HSF1 activation and the induction of the heat shock response, while the reassociation of HSP90 with HSF1 is essential for terminating the response. According to this model, disrupting HSF1–HSP90 interaction by inhibiting HSP90 leads to enhanced HSF1 activation and, consequently, increased compensatory synthesis of heat shock proteins, including HSP70, HSP27, and HSP40 [50,51].

Understanding the interaction mechanisms between HSF1 and HSP90 is crucial for investigating the regulation of HSF1 and the compensatory induction of heat shock proteins resulting from impaired HSF1–HSP90 interactions. In this regard, Kijima et al. demonstrated that the stress-inducible isoform HSP90α binds more strongly to HSF1 than the constitutively expressed HSP90β. In addition, HSP90 must be in a “closed” conformation, where its N-terminal domains (NTDs) are bound to ATP, which promotes temporary dimerization of HSP90 [52]. It has also been shown that the binding efficiency of HSF1 to HSP90 is affected by the phosphorylation status of HSF1. For instance, MAPK-activated protein kinase 2 (MAPK2) phosphorylates HSF1 at serine 121, inhibiting its binding to HSE in HSP gene promoters by enhancing its binding affinity for HSP90 [53].

**HSP70** is an important regulator of HSF1 activity and therefore plays a significant role in the induction of HSR. Early studies revealed that overexpression of HSP70 negatively affects HSF1 and HSR activation, and accordingly, HSP70 inhibition can lead to compensatory synthesis of heat shock proteins [54,55]. Furthermore, when the HSF1 trimer binds to DNA and activates transcription, HSP70, in conjunction with DNAJB1, interacts with the transactivation domain of HSF1 to attenuate its transcriptional activity (Figure 2). HSP70 and DNAJB1 also bind to the HR-B proximal binding site of HSF1, leading to the disassembly of the factor’s trimer from the HSE and the monomerization of HSF1 [56]. Thus, HSP70 induction is required to suppress HSF1 activity, and following heat shock, HSF1 is rebounded by HSP70, and its transcriptional activity is again repressed [55,57].

**TRiC** (T-complex protein-1 ring complex, also known as CCT) is a chaperonin that facilitates the folding of target proteins through ATP-dependent conformational changes [58]. It was demonstrated that the central ATP-dependent chaperonin complex TRiC/CCT regulates HSF1 activity through direct interaction. Notably, a small molecule activator of HSF1, known as HSF1A, has been found to protect cells from stress-induced apoptosis by binding to TRiC subunits and inhibiting its activity, thereby triggering HSF1 activation both in vitro and in vivo [59].

**CHIP** (The carboxy-terminus of HSP70-interacting protein) functions as a ubiquitin ligase and a co-chaperone, playing a pivotal role in cancer progression [60]. Initial studies showed that CHIP regulates HSR activation by facilitating trimerization, nuclear translocation, and transcriptional activation of HSF1, thereby protecting against stress-induced apoptosis in fibroblasts [61]. Afterward, Kim et al. demonstrated that HSF1 directly interacts with CHIP through its N-terminal region, which was induced by heat stress in non-malignant cells [62]. Moreover, CHIP has been shown to stabilize HSF1, facilitating its nuclear translocation and activity to protect cardiomyocytes under doxorubicin exposure [63]. Although such mechanisms have not yet been described in malignant cells, the above-demonstrated involvement of CHIP in the regulation of stress-induced HSF1 activation may also play an important role in the enhanced activity of the factor during oncogenesis.

**BAG** (BCL-2-associated athanogens) family members are involved in a variety of cellular processes such as autophagy, apoptosis, and maintenance of protein homeostasis [64]. Acting as HSP70’s co-chaperones, BAG proteins are also implicated in numerous pro-oncogenic PPIs. Under heat stress, BAG3 has been identified as a main regulator of nucleocytoplasmic shuttling of HSF1 in both cancer and normal cells [65]. Another member of the BAG family, BAG1, has been demonstrated to promote the phosphorylation of HSF1, implying a potential role in regulating HSF1 transcriptional activity by HSPs. However, despite this implication, there is currently no evidence of a direct interaction between HSF1 and BAG1 [66].

### 3.2. HSF1-Modifying Enzymes as Potent Regulators of Its Activity

During its journey to the functional site in the nucleus, HSF1 molecules are subjected to a variety of post-translational modifications [67]. Most of these events are primarily mediated by kinases targeting specific amino acid residues on HSF1 molecules that change the conformation of the whole polypeptide. Notably, kinases and their substrate targets are highly specific, impacting HSF1 maturation, intranuclear transport, and also transcriptional activity. In addition to phosphorylation, other modifications such as acetylation and SUMOylation have been shown to influence HSF1 activation [68,69]. In the following section, we present data on the enzymes catalyzing these modifications and their effects on HSF1 function.

**AKT** (also known as protein kinase B or PKB) is a well-known serine/threonine kinase of the PI3K/AKT/mTOR signaling pathway [70]. In tumor cells, AKT is involved in the regulation of proliferation, migration, and metabolic regulation. As previously described, HSF1 transcriptional activity is closely linked to its trimerization and phosphorylation. Carpenter et al. showed that AKT physically interacts with HSF1 to activate it, leading to subsequent activation of Slug, the EMT program, and the progression of breast cancer [24]. In addition, Lu et al. investigated the effect of AKT1 on HSF1 activation and found that AKT1 phosphorylates multiple sites in HSF1 at once, stimulating its transcriptional activity. Specifically, phosphorylation at T142 enhances HSF1 trimerization, while phosphorylation at S230, S326, and T527 enhances HSF1’s interaction with CDK9 (a component of positive transcription elongation factor, P-TEFb) and TFIIB, thus promoting transcription [71] (Figure 2). It was found that co-activation of AKT and HSF1 occurs in breast cancer and that their combined targeting using selective inhibitors exhibited a synergistic effect on tumor cells [72]. Furthermore, the inhibition of HSF1 in human hepatocarcinoma cells resulted in a reduction in the PI3K/AKT/mTOR signaling pathway and the associated metabolic pathways [73].

**mTOR** (the mammalian target of rapamycin) is a serine/threonine kinase, which is a part of two large complexes—mTORC1 and mTORC2. mTORC1 is a rapamycin-sensitive complex consisting of six proteins that sense various stress signals, including protein misfolding, growth factor deprivation, energy deficiency, hypoxia, and amino acid shortage, and also plays a crucial role in promoting cellular anabolism and autophagy [74,75]. mTORC2 is relatively less sensitive to rapamycin and primarily regulates cell survival and metabolism. Both complexes are involved in the regulation of HSF1. The major role of mTORC1 in regulating HSF1 activity is its ability to phosphorylate HSF1 at S326 following exposure to stress [76]. Additionally, the mTORC1/HSF1 axis is important for β-catenin and HuR expression in mammalian cells [25]. Moreover, Lu et al. discovered that, in addition to phosphorylation at S326, mTORC1 can also phosphorylate three other residues, S221, S241, and S344, all located in the regulatory domain of HSF1. The functional significance of these phosphorylation events for the regulation of HSF1 activity remains unclear [71]. It is noteworthy that HSF1 exerts its influence on mTORC1 activity and translation by inactivating and sequestering JNK, thereby promoting stress resistance and growth [31]. In colorectal cancer cells, HSF1 was also found to stimulate glutaminase 1 (GLS1)-dependent mTOR activation by recruiting DNMT3a to epigenetically repress microRNA137 (MIR137) expression that targets GLS1 [77]. mTORC2 is also involved in the regulation of HSF1, but its influence is much less. Holmes et al. showed that in human glioblastoma cells, mTORC2 activates AKT1 via phosphorylation, which then phosphorylates HSF1 at S326. In turn, activated HSF1 by HuR elevates Rictor expression, resulting in enhanced mTORC2 activity in tumor cells [39].

**MEK** and **ERK** are protein kinases that form a central part of the RAS-RAF-MEK-ERK signaling pathway, which is associated with the regulation of cell proliferation and metabolism [78]. Deregulation of this pathway is commonly observed during carcinogenesis, with this cascade playing a central role in the maintenance of several cancers, including melanoma, pancreatic cancer, lung cancer, colorectal cancer, and breast cancer [79]. MEK is a kinase with dual specificity, i.e., it can phosphorylate both serine/threonine and tyrosine residues. For a long time, ERK1/2 was thought to be the only target phosphorylated by MEK. However, Tang et al. found that heat shock activates RAS/MAPK signaling and induces physical interactions between MEK and HSF1, resulting in the phosphorylation of HSF1 by S326 (Figure 2) as well as its nuclear translocation [80]. The involvement of ERK1/2 in the regulation of HSF1 activity is multifaceted. On the one hand, it has been reported that ERK1/2 phosphorylates HSF1 at Ser307, a modification that exerts a constitutive repressive effect under basal growth conditions. In addition, the authors demonstrated that ERK indirectly represses HSF1 activation through a negative feedback mechanism. ERK phosphorylates MEK at Thr292/386, thereby inhibiting MEK-mediated phosphorylation of S326 on HSF1. However, ERK can also directly phosphorylate HSF1 at S326, activating it and promoting drug resistance of cancer cells [81].

**AMPK** (5′-Adenosine monophosphate-activated protein kinase) serves as a primary sensor of cellular energy and also functions as a negative regulator of HSF1. Upon metabolic stress, AMPK physically interacts with and phosphorylates HSF1 at S121 (Figure 2), which impairs the nuclear translocation and stability of HSF1 [82]. At the same time, HSF1 exerts reciprocal regulation on AMPK, shifting it into an inactive conformation. Dai and colleagues suggest that under non-stressed conditions, a large portion of cellular AMPK interacts with inactive, monomeric HSF1, forming a mutually repressive complex. A smaller fraction of AMPK remains free from HSF1 and available for activation. Such interaction favors the participation of HSF1 in regulating the processes of lipogenesis, cholesterol synthesis, and protein cholesteroylation. Additionally, HSF1’s influence on AMPK contributes to the control of body fat mass in vivo and also regulates the lipogenic phenotype and growth of melanomas [83]. In a study by Chen et al., a correlation was observed between elevated levels of active HSF1 and reduced levels of phosphorylated AMPK (p-AMPK) in pancreatic adenocarcinoma cells, which promoted EMT, invasion, and overall tumor progression. Conversely, the activation of AMPK, both in vitro and in vivo, was found to inhibit HSF1 activity and contributed to a decrease in tumor progression rates [84].

### 3.3. Interactions Regulating HSF1-Dependent Transcription

HSF1 exerts direct or indirect control over nearly 1300 genes, a regulatory mechanism that entails both the activation of certain genes and the inhibition of others [85]. Such extensive transcriptional regulation is feasible due to HSF1 interactions with a large variety of proteins. Here, we review some of the major complexes that are essential for maintaining HSF1-dependent transcriptional activity and cell viability.

**c-MYC** is a nuclear transcription factor that regulates growth, metabolism, and cell death. Constitutive aberrant activation of this factor is observed in approximately 70% of tumors. MYC proteins form heterodimers with MYC-associated factor X (MAX) protein, which typically binds to a canonical DNA sequence element, the E-box, in the promoter regions of several MYC target genes. In 2023, Xu et al. reported that HSF1 interacts with c-MYC/MAX complexes under non-stress conditions, thereby promoting c-MYC’s transcriptional activity [86].

**TRRAP/TIP60** is a histone acetyltransferase complex that is involved in the post-translational modification of histones, and thus, has profound effects on chromatin structure within the eukaryotic nucleus. TRRAP was initially discovered as a coactivator for the c-MYC and E2F transcription factors, exhibiting an essential role in their pro-oncogenic activity [87]. Fujimoto et al. found that HSF1 recruits the TRRAP-TIP60 complex and p300 during heat shock, which acetylates histones and promotes acetylation-dependent monoubiquitination of H2B by TRIM33 and TRIM24. This leads to the establishment of an active chromatin state in the promoter region of the HSP genes. The recruitment of these histone-modifying enzymes is triggered by the phosphorylation of HSF1 at S419 via PLK1 and is associated with enhanced [88].

**PARP1** (poly(ADP-ribose) polymerase 1) is a multifunctional regulator of chromatin structure, transcription, and DNA repair, due to its ability to carry out ADP-ribosylation of histones, while PARP13 acts as a scaffold protein for HSF1 and PARP1 interaction. The PARP1-HSF1-PARP13 triple complex regulates DNA damage-induced gene expression. In response to DNA damage, PARP1 dissociates from the complex, and HSF1-PARP13 regulates PARP1 redistribution to sites of DNA repair. Thus, this complex protects cells from genotoxic stress and promotes breast cancer progression [89]. In a subsequent study, the authors explored the role of this complex during heat shock and revealed that, prior to stress exposure, PARP1 occupies HSEs domains at the HSP70 promoter and interacts with the HSF1-PARP13 complex [90]. Upon heat shock, PARP1 undergoes auto-poly(ADP-ribosyl)ation (auto-PARylation), leading to its dissociation from HSF1-PARP13 and redistribution to the HSP70 loci. This event, in turn, facilitates the establishment of an active chromatin state, enabling HSF1 to bind to the promoter region of HSP70 and activate HSR. The authors also found that phosphorylation of HSF1 at S121 is necessary for the successful dissociation of PARP13 from the complex during the initial steps of HSR activation [90]. In addition, pretreatment with metformin, an AMPK activator, averts HSR activation partly by blocking the assembly of the ternary complex.

**PRMT5** is an arginine methyltransferase responsible for catalyzing the methylation of the guanidine nitrogen atoms of arginine residues, using S-adenosylmethionine (SAM) as a methyl donor. Huang et al. revealed that HSF1 expression is upregulated in breast cancer cells and that HSF1 interacts with PRMT5, influencing histone methylation and increasing the expression of several oncogenes. Activation of this HSF1-PRMT5 axis in the cell promotes EMT and proliferation of metastatic cells in lymph nodes. In addition, HSF1 also enhances CCL20 secretion by tumor cells, facilitating macrophage infiltration [91].

**HSF2** is a protein that also belongs to the heat shock factor family, but unlike HSF1, its functions in stress responses and other cellular processes remain incompletely understood. It is known that HSF2 is essential for normal development, including spermatogenesis and brain development. At the same time, accumulating evidence points to its involvement in tumors. For example, HSF2 is required to prevent the progression of prostate cancer [92]. Conversely, HSF2 was recently reported to physically interact with HSF1 in several types of cancer, and this interaction promotes the expression of HSPs and non-HSP transcriptional targets to support tumor malignancy [93].

**p53** is one of the key regulators of the cellular stress response, and it is not surprising that its interaction with HSF1 is necessary to coordinate this response. The reciprocal regulation between HSF1 and p53 occurs at multiple levels simultaneously, underscoring the importance of this interaction for cellular functions. For instance, in response to genotoxic stress, HSF1 is required for p53-mediated transcription and, in complex with p53, regulates the dynamic changes in p21 expression, a direct target of the p53 protein. Furthermore, under chemotherapy, HSF1 can form a complex with the DNA damage kinases, ATR and Chk1, to regulate p53 phosphorylation [40]. UV-induced stress also leads to HSF1 activation and its interaction with p53 [94]. HSF1 also affects p53 activity through HSF1-dependent chaperones, which can bind with wt and mutant forms of p53. These interactions stabilize the multi-chaperone complexes [95,96], thereby extending the half-life of p53 in cancer cells [97,98]. In another paper on the interaction between these two proteins, the authors describe a mechanism in which HSF1 plays a role in the loss of wild-type p53 and the selection for mutant p53 (mut-p53), contributing to tumor progression [99]. This is partly due to the absence of the inhibitory effect of wild-type p53 on HSF1 [100,101]. Mut-p53 promotes HSF1 phospho-activation and stabilization by activating the EGFR/ErbB2/MAPK/PI3K signaling cascade. In addition, mut-p53 directly interacts with HSF1-S326 to recruit HSF1 to its specific DNA binding sites on target gene promoters, thereby enhancing its transcriptional activity.

**Table 1:** Summary of major HSF1 interactors and their role in the regulation of HSF1 activity.

Interacting Protein	Characteristics of PPI	Cell Lines	Methods	Outcomes of the Interaction with HSF1	References
HSP90	HSP90 in its “closed” conformation directly binds to the HR-A/B domain of HSF1		Co-IP	negative regulation of HSF1 activity	[49,52,102]
HSP70	HSP70 interacts directly through the TAD and HR-B domains of HSF1	HeLa	Co-IP; GST in vitro binding assay	negative regulation of HSF1 activity	[47,56]
HSP40	HSP40 interacts directly through the TAD and HR-B domains of HSF1	HeLa	GST in vitro binding assays	negative regulation of HSF1 activity	[47,56]
TRiC/CCT	direct interaction	HeLa, HEK293T, NIH3T3, MEFs	co-IP, in vitro binding assays	negative regulation of HSF1 activity	[59]
CHIP	direct interaction through NTD of HSF1	COS7, HEK 293, H9c2, NRVMs	co-IP	stabilizing HSF1 and facilitating its nuclear translocation, which protects against stress-induced apoptosis	[61,62,63]
BAG-1	no direct interaction was demonstrated	BT-474, MCF-7	In silico predictions of protein–protein interactions	no direct interaction was demonstrated	[66]
BAG-3	interaction through BAG-domain	HeLa	co-IP	regulation of HSF1 nuclear shuttling upon heat stress	[65]
AKT1	directly interacts with HSF1 and phosphorylates HSF1 at S326 mediates multiple phosphorylations of HSF1	BT-474, MCF-7, MDA-MB-231, HEK293, MCF-7	IP-WB, cell-free kinase assay	Slug overexpression and EMT Regulation of HSF1 transcriptional activity	[24,71]
mTORC1	transient interaction	HeLa	in vitro kinase assay	during proteotoxic stress activates HSF1 by phosphorylation at S326	[76]
MEK	physically interacts with HSF1	HEK293T, HeLa	co-IP	preserves proteostasis by activating HSF1	[80]
ERK1/2	physically interacts with HSF1 (immediately after heat shock)	HEK293T, HeLa	co-IP	suppresses MEK-HSF1 interactions to inactivate HSF1	[80]
AMPK	physically interacts with HSF1 and phosphorylates it at S121	NIH3T3, HEK293T, MEF	co-IP, in vitro kinase assays	HSF1 inactivation that impacts stress resistance, proteostasis, and malignant growth. HSF1 governs lipid metabolism and protein cholesteroylation through AMPK regulation	[82]
c-MYC	forms a complex with HSF1 and MAX	MEF, HEK293T, HeLa	Lumit immunoassays, co-IP, proximity ligation assay	HSF1 activates c-MYC transcriptional activity via GCN5	[86]
TRRAP-TIP60	forms a complex with HSF1	MEF	ChIP-MS, co-IP	promotes tumorigenesis	[88]
PARP1-PARP13	forms a complex with HSF1	HEK293, HeLa	ChIP seq, IP-WB	protects cells from genotoxic stress and promotes breast cancer progression	[89]
PRMT5	forms a complex with HSF1	T24	co-IP, immunofluorescence	influences on histone methylation; increases the expression of a number of oncogenes; EMT and proliferation of metastatic cells in lymph nodes	[91]
HSF2	physically interacts with HSF1	MCF-7, ZR-75-1, 231, PC3M, NCI-H838	LUMIER assay, IP-MS, IP	promotes the expression of HSPs and non-HSP transcriptional targets to support malignant features	[93]
p53	during genotoxic stress and UV forms a complex with HSF1	HEK293T, U2OS, MEF	co-IP	regulates the expression of p21	[33]

## 4. Perspectives on Modulating HSF1 via Its Interactors

Efforts to develop drugs targeting HSF1 have been ongoing in preclinical studies for an extended long period, but none have yet reached clinical application. This is largely attributable to the absence of a definitive HSF1 ligand, potential target sites within its tertiary structure, as well as the complexity of its activation cycle and the diverse post-translational modifications. In addition, HSF1 is an essential factor for the survival of normal cells under stress conditions, indicating that any potential inhibitor must be tumor cell-specific. Despite these challenges, more than 20 HSF1 inhibitors, of both synthetic and natural origin, have been reported. However, common issues with these compounds include either high toxicity or limited knowledge about their mechanisms of action and specificity. Currently, only the bisamide compound, CCT361814, also known as NXP800, has advanced to Phase I clinical trials. This inhibitor of HSF1 induced tumor regression in human ovarian adenocarcinoma xenografts and was more effective than carboplatin in inhibiting the proliferation of ovarian cancer cells [103].

The primary goal of this review is to highlight the multiple interactions between HSF1 and its partners, with the hope that by modulating such interplays, HSF1 activity in cancer cells can be efficiently targeted. At present, the search for PPI disruptors is progressing rapidly. One of the notable examples is the discovery of JG-98, a compound introduced by Jason Gestwicki [104], which dissociates the interaction between HSP70 and HMGB1, thereby effectively inhibiting relapse in pseudodormant cancer cell populations [105].

Among the PPIs discussed in the review, the interactions between HSF1 and chaperones, particularly HSP90, are the most robust and have been the subject of extensive clinical investigation to date. A common feature of most HSP90 inhibitors is that they primarily target the N-terminal domain of the HSP90 molecule. Such inhibitors disrupt the ATPase activity of HSP90, causing conformational changes in the homodimer and thus preventing its folding activity. Unfortunately, none of these inhibitors have been approved for clinical use, with most failing due to issues like drug resistance, toxicity that limits the dosage, and unfavorable pharmacokinetic profiles [106]. Apparently, a significant limitation in targeting HSP90 in antitumor therapy is its inhibitory effect on HSF1, whereby HSP90 inhibition can trigger feedback mechanisms leading to the upregulation of HSP27, HSP40, HSP70, and HSP90 [107,108]. For instance, onalespib (AT13387), a second-generation small molecule inhibitor of Hsp90 that acts as an ATP competitor at the N-terminal domain of Hsp90, has been shown to promote HSR and result in increased expression of HSP70, HSP27, and other pro-survival proteins [109], contributing to its failure in Phase II clinical trials. In addition, Pesonen et al. demonstrated that gambogic and gambogenic acids are able to disrupt the complex between HSP90 and HSF1, which, in turn, leads to the induction of a robust HSF1-dependent HSR [110]. Targeting and developing inhibitors for the middle domain (MD) and carboxyterminal domain (CTD) of HSP90 may offer a more beneficial therapeutic strategy, as these approaches could bypass the HSR activation. However, there is currently no information on how MD or CTD inhibitors affect the interaction between HSP90 and HSF1 [111].

The use of compounds to disrupt the interactions between the kinases described above and HSF1 appears to be the most promising approach for modulating HSF1 activity, as these kinases are responsible for fine-tuning their regulation through post-translational modifications. For example, metformin, a well-known AMPK activator, has been shown to suppress HSF1 transcriptional activity and induce proteotoxic stress in tumor cells, thereby inhibiting tumor growth [82]. Interestingly, recent studies have shown that metformin exhibits a higher affinity for HSF1 than AMPK in vitro, suggesting that HSF1 may be a more sensitive target of metformin [112]. Investigations into the effect of rapamycin, an mTOR inhibitor, on HSF1 activity have demonstrated conflicting results, which seem to depend on the dosage of the drug used [71,76,82]. In addition, other studies have reported that the use of the pan-AKT inhibitor MK-2206 leads to a loss of HSF1 activity in response to heat stress [71,113]

However, targeting the dissociation of HSF1 from its associated kinases has several significant challenges, with only ERK activators emerging as the most promising approach. First, ERK appears to be less prone to mutation compared to other kinases, and prior to the development and clinical use of small molecule ERK inhibitors, mutations of ERK were virtually unknown in human cancers. In addition, another issue in targeting kinases is the lack of highly selective modulators that do not affect other enzymes with similar specificities. Nevertheless, targeting ERK remains a promising strategy since it is positioned at the lower end of the signaling cascade, which minimizes its impact on other critical pathways, unlike the previously discussed kinases in this review. Furthermore, another reason for choosing this approach is that human cells, including cancer cells, exhibit poor tolerance for excessive ERK activity levels. Given that ERK is a downstream target of the RAS-RAF-MEK-ERK signaling cascade, inhibiting upstream molecules may lead to reduced ERK activity and subsequent activation of HSF1, as ERK exerts negative regulation on HSF1. In this context, maintaining ERK in an active state may represent an additional antitumor strategy, which is emerging as a relevant area of research [114]. An example of the efficacy of the hyperactivation of ERK in tumor cells is demonstrated by the compound ACA-28 [115], although data regarding its impact, or that of similar compounds, on HSF1 activity is currently lacking in the literature.

## 5. Conclusions

HSF1 is a multifaceted regulator of cellular physiology known to possess a great protective capacity due to its role in driving the expression of heat shock proteins. HSP90, HSP27, and particularly HSP70 were found to elicit protective potential against a wide range of stressors and harmful conditions, a property already leveraged in therapies for various diseases, tissue transplantation, and cardiovascular diseases. However, in cancer cells, the protective activity of HSPs becomes detrimental, as HSPs that possess chaperonic activity are known to impede apoptosis and even to promote the cell cycle, thereby contributing to cancer cell survival and tumor progression. Therefore, agents capable of repressing the expression of HSP chaperones, like HSF1 inhibitors, are of particular interest for cancer therapy, as many tumors are known to highly rely on the chaperonic apparatus and express elevated levels of HSPs. At the same time, and as highlighted in this review, the development of new HSF1 inhibitors should also focus on disrupting its interactions with key proteins that influence its activation, and therefore, such “delicate” molecules could presumably represent a more effective and promising strategy for regulating HSF1 activity in antitumor therapies.

## References

[1] Mayer M.P. (2024). Hsf1 and Hsf2 in Normal, Healthy Human Tissues: Immunohistochemistry Provokes New Questions. Cell Stress Chaperones.

[2] Mendillo M.L., Santagata S., Koeva M., Bell G.W., Hu R., Tamimi R.M., Fraenkel E., Ince T.A., Whitesell L., Lindquist S. (2012). HSF1 Drives a Transcriptional Program Distinct from Heat Shock to Support Highly Malignant Human Cancers. Cell.

[3] Kabakov A., Yakimova A., Matchuk O. (2020). Molecular Chaperones in Cancer Stem Cells: Determinants of Stemness and Potential Targets for Antitumor Therapy. Cells.

[4] Powell C.D., Paullin T.R., Aoisa C., Menzie C.J., Ubaldini A., Westerheide S.D. (2016). The Heat Shock Transcription Factor HSF1 Induces Ovarian Cancer Epithelial-Mesenchymal Transition in a 3D Spheroid Growth Model. PLoS ONE.

[5] Joutsen J., Pessa J.C., Jokelainen O., Sironen R., Hartikainen J.M., Sistonen L. (2024). Comprehensive Analysis of Human Tissues Reveals Unique Expression and Localization Patterns of HSF1 and HSF2. Cell Stress Chaperones.

[6] Roos-Mattjus P., Sistonen L. (2022). Interplay between Mammalian Heat Shock Factors 1 and 2 in Physiology and Pathology. FEBS J..

[7] Neudegger T., Verghese J., Hayer-Hartl M., Hartl F.U., Bracher A. (2016). Structure of Human Heat-Shock Transcription Factor 1 in Complex with DNA. Nat. Struct. Mol. Biol..

[8] Gomez-Pastor R., Burchfiel E.T., Thiele D.J. (2018). Regulation of Heat Shock Transcription Factors and Their Roles in Physiology and Disease. Nat. Rev. Mol. Cell Biol..

[9] Puustinen M.C., Sistonen L. (2020). Molecular Mechanisms of Heat Shock Factors in Cancer. Cells.

[10] Dai C. (2018). The Heat-Shock, or HSF1-Mediated Proteotoxic Stress, Response in Cancer: From Proteomic Stability to Oncogenesis. Philos. Trans. R. Soc. Lond. B Biol. Sci..

[11] Dayalan Naidu S., Sutherland C., Zhang Y., Risco A., de la Vega L., Caunt C.J., Hastie C.J., Lamont D.J., Torrente L., Chowdhry S. (2016). Heat Shock Factor 1 Is a Substrate for P38 Mitogen-Activated Protein Kinases. Mol. Cell. Biol..

[12] Moreno R., Banerjee S., Jackson A.W., Quinn J., Baillie G., Dixon J.E., Dinkova-Kostova A.T., Edwards J., de la Vega L. (2021). The Stress-Responsive Kinase DYRK2 Activates Heat Shock Factor 1 Promoting Resistance to Proteotoxic Stress. Cell Death Differ..

[13] Chin Y., Gumilar K.E., Li X.G., Tjokroprawiro B.A., Lu C.H., Lu J., Zhou M., Sobol R.W., Tan M. (2023). Targeting HSF1 for Cancer Treatment: Mechanisms and Inhibitor Development. Theranostics.

[14] Chu B., Soncin F., Price B.D., Stevenson M.A., Calderwood S.K. (1996). Sequential Phosphorylation by Mitogen-Activated Protein Kinase and Glycogen Synthase Kinase 3 Represses Transcriptional Activation by Heat Shock Factor-1. J. Biol. Chem..

[15] Kline M.P., Morimoto R.I. (1997). Repression of the Heat Shock Factor 1 Transcriptional Activation Domain Is Modulated by Constitutive Phosphorylation. Mol. Cell. Biol..

[16] Kourtis N., Lazaris C., Hockemeyer K., Balandrán J.C., Jimenez A.R., Mullenders J., Gong Y., Trimarchi T., Bhatt K., Hu H. (2018). Oncogenic hijacking of the stress response machinery in T cell acute lymphoblastic leukemia. Nat. Med..

[17] Hoj J.P., Mayro B., Pendergast A.M. (2020). The ABL2 Kinase Regulates an HSF1-Dependent Transcriptional Program Required for Lung Adenocarcinoma Brain Metastasis. Proc. Natl. Acad. Sci. USA.

[18] Scherz-Shouval R., Santagata S., Mendillo M.L., Sholl L.M., Ben-Aharon I., Beck A.H., Dias-Santagata D., Koeva M., Stemmer S.M., Whitesell L. (2014). The Reprogramming of Tumor Stroma by HSF1 Is a Potent Enabler of Malignancy. Cell.

[19] Shi Y., Sun L., Zhang R., Hu Y., Wu Y., Dong X., Dong D., Chen C., Geng Z., Li E. (2021). Thrombospondin 4/Integrin A2/HSF1 Axis Promotes Proliferation and Cancer Stem-like Traits of Gallbladder Cancer by Enhancing Reciprocal Crosstalk between Cancer-Associated Fibroblasts and Tumor Cells. J. Exp. Clin. Cancer Res..

[20] Hockemeyer K., Sakellaropoulos T., Chen X., Ivashkiv O., Sirenko M., Zhou H., Gambi G., Battistello E., Avrampou K., Sun Z. (2024). The Stress Response Regulator HSF1 Modulates Natural Killer Cell Anti-Tumour Immunity. Nat. Cell Biol..

[21] Jacobs C., Shah S., Lu W.C., Ray H., Wang J., Hockaden N., Sandusky G., Nephew K.P., Lu X., Cao S. (2024). HSF1 Inhibits Antitumor Immune Activity in Breast Cancer by Suppressing CCL5 to Block CD8+ T-Cell Recruitment. Cancer Res..

[22] Komarova E.Y., Marchenko L.V., Zhakhov A.V., Nikotina A.D., Aksenov N.D., Suezov R.V., Ischenko A.M., Margulis B.A., Guzhova I.V. (2020). Extracellular Hsp70 Reduces the Pro-Tumor Capacity of Monocytes/Macrophages Co-Cultivated with Cancer Cells. Int. J. Mol. Sci..

[23] Nikotina A.D., Vladimirova S.A., Kokoreva N.E., Nevdakha V.A., Lazarev V.F., Kuznetcova L.S., Komarova E.Y., Suezov R.V., Efremov S., Leonova E. (2024). Novel Mechanism of Drug Resistance Triggered by Tumor-Associated Macrophages through Heat Shock Factor-1 Activation. Cancer Immunol. Immunother..

[24] Carpenter R.L., Paw I., Dewhirst M.W., Lo H.W. (2015). Akt Phosphorylates and Activates HSF-1 Independent of Heat Shock, Leading to Slug Overexpression and Epithelial-Mesenchymal Transition (EMT) of HER2-Overexpressing Breast Cancer Cells. Oncogene.

[25] Chou S.D., Murshid A., Eguchi T., Gong J., Calderwood S.K. (2015). HSF1 Regulation of β-Catenin in Mammary Cancer Cells through Control of HuR/ElavL1 Expression. Oncogene.

[26] Li Y., Xu D., Bao C., Zhang Y., Chen D., Zhao F., Ding J., Liang L., Wang Q., Liu L. (2015). MicroRNA-135b, a HSF1 target, promotes tumor invasion and metastasis by regulating RECK and EVI5 in hepatocellular carcinoma. Oncotarget.

[27] Prince T.L., Lang B.J., Guerrero-Gimenez M.E., Fernandez-Muñoz J.M., Ackerman A., Calderwood S.K. (2020). HSF1: Primary Factor in Molecular Chaperone Expression and a Major Contributor to Cancer Morbidity. Cells.

[28] Xi C., Hu Y., Buckhaults P., Moskophidis D., Mivechi N.F. (2012). Heat Shock Factor Hsf1 Cooperates with ErbB2 (Her2/Neu) Protein to Promote Mammary Tumorigenesis and Metastasis. J. Biol. Chem..

[29] Stankiewicz A.R., Livingstone A.M., Mohseni N., Mosser D.D. (2009). Regulation of Heat-Induced Apoptosis by Mcl-1 Degradation and Its Inhibition by Hsp70. Cell Death Differ..

[30] Li C.Y., Lee J.S., Ko Y.G., Kim J.I., Seo J.S. (2000). Heat Shock Protein 70 Inhibits Apoptosis Downstream of Cytochrome c Release and Upstream of Caspase-3 Activation. J. Biol. Chem..

[31] Edlich F., Erdmann F., Jarczowski F., Moutty M.C., Weiwad M., Fischer G. (2007). The Bcl-2 Regulator FKBP38-Calmodulin-Ca2+ Is Inhibited by Hsp90. J. Biol. Chem..

[32] Li D., Marchenko N.D., Schulz R., Fischer V., Velasco-Hernandez T., Talos F., Moll U.M. (2011). Functional Inactivation of Endogenous MDM2 and CHIP by HSP90 Causes Aberrant Stabilization of Mutant P53 in Human Cancer Cells. Mol. Cancer Res..

[33] Logan I.R., Mcneill H.V., Cook S., Lu X., Meek D.W., Fuller-Pace F.V., Lunec J., Robson C.N. (2009). Heat Shock Factor-1 Modulates P53 Activity in the Transcriptional Response to DNA Damage. Nucleic Acids Res..

[34] Schulz R., Streller F., Scheel A.H., Rüschoff J., Reinert M.C., Dobbelstein M., Marchenko N.D., Moll U.M. (2014). HER2/ErbB2 Activates HSF1 and Thereby Controls HSP90 Clients Including MIF in HER2-Overexpressing Breast Cancer. Cell Death Dis..

[35] Wang B., Lee C.W., Witt A., Thakkar A., Ince T.A. (2015). Heat Shock Factor 1 Induces Cancer Stem Cell Phenotype in Breast Cancer Cell Lines. Breast Cancer Res. Treat..

[36] Kusumoto H., Hirohashi Y., Nishizawa S., Yamashita M., Yasuda K., Murai A., Takaya A., Mori T., Kubo T., Nakatsugawa M. (2018). Cellular Stress Induces Cancer Stem-like Cells through Expression of DNAJB8 by Activation of Heat Shock Factor 1. Cancer Sci..

[37] Yasuda K., Hirohashi Y., Mariya T., Murai A., Tabuchi Y., Kuroda T., Kusumoto H., Takaya A., Yamamoto E., Kubo T. (2017). Phosphorylation of HSF1 at Serine 326 Residue Is Related to the Maintenance of Gynecologic Cancer Stem Cells through Expression of HSP27. Oncotarget.

[38] Su K.H., Cao J., Tang Z., Dai S., He Y., Sampson S.B., Benjamin I.J., Dai C. (2016). HSF1 Critically Attunes Proteotoxic Stress Sensing by MTORC1 to Combat Stress and Promote Growth. Nat. Cell Biol..

[39] Holmes B., Benavides-Serrato A., Freeman R.S., Landon K.A., Bashir T., Nishimura R.N., Gera J. (2018). MTORC2/AKT/HSF1/HuR Constitute a Feed-Forward Loop Regulating Rictor Expression and Tumor Growth in Glioblastoma. Oncogene.

[40] Desai S., Liu Z., Yao J., Patel N., Chen J., Wu Y., Ahn E.E.Y., Fodstad O., Tan M. (2013). Heat Shock Factor 1 (HSF1) Controls Chemoresistance and Autophagy through Transcriptional Regulation of Autophagy-Related Protein 7 (ATG7). J. Biol. Chem..

[41] Watanabe Y., Tsujimura A., Taguchi K., Tanaka M. (2017). HSF1 Stress Response Pathway Regulates Autophagy Receptor SQSTM1/P62-Associated Proteostasis. Autophagy.

[42] Koh G.C.K.W., Porras P., Aranda B., Hermjakob H., Orchard S.E. (2012). Analyzing Protein-Protein Interaction Networks. J. Proteome Res..

[43] Rodina A., Wang T., Yan P., Gomes E.D., Dunphy M.P.S., Pillarsetty N., Koren J., Gerecitano J.F., Taldone T., Zong H. (2016). The Epichaperome Is an Integrated Chaperome Network That Facilitates Tumour Survival. Nature.

[44] Cheng S.S., Yang G.J., Wang W., Leung C.H., Ma D.L. (2020). The Design and Development of Covalent Protein-Protein Interaction Inhibitors for Cancer Treatment. J. Hematol. Oncol..

[45] Bagchi A. (2018). Protein-Protein Interactions: Basics, Characteristics, and Predictions. Soft Comput. Biol. Syst..

[46] Burchfiel E.T., Vihervaara A., Guertin M.J., Gomez-Pastor R., Thiele D.J. (2021). Comparative Interactomes of HSF1 in Stress and Disease Reveal a Role for CTCF in HSF1-Mediated Gene Regulation. J. Biol. Chem..

[47] Shi Y., Mosser D.D., Morimoto R.I. (1998). Molecular Chaperones as HSF1-Specific Transcriptional Repressors. Genes Dev..

[48] Masser A.E., Ciccarelli M., Andréasson C. (2020). Hsf1 on a Leash—Controlling the Heat Shock Response by Chaperone Titration. Exp. Cell Res..

[49] Zou J., Guo Y., Guettouche T., Smith D.F., Voellmy R. (1998). Repression of Heat Shock Transcription Factor HSF1 Activation by HSP90 (HSP90 Complex) That Forms a Stress-Sensitive Complex with HSF1. Cell.

[50] Voellmy R., Boellmann F. (2007). Chaperone Regulation of the Heat Shock Protein Response. Adv. Exp. Med. Biol..

[51] Whitesell L., Bagatell R., Falsey R. (2005). The Stress Response: Implications for the Clinical Development of Hsp90 Inhibitors. Curr. Cancer Drug Targets.

[52] Kijima T., Prince T.L., Tigue M.L., Yim K.H., Schwartz H., Beebe K., Lee S., Budzynski M.A., Williams H., Trepel J.B. (2018). HSP90 Inhibitors Disrupt a Transient HSP90-HSF1 Interaction and Identify a Noncanonical Model of HSP90-Mediated HSF1 Regulation. Sci. Rep..

[53] Wang X.Z., Khaleque M.A., Mei J.Z., Zhong R., Gaestel M., Calderwood S.K. (2006). Phosphorylation of HSF1 by MAPK-Activated Protein Kinase 2 on Serine 121, Inhibits Transcriptional Activity and Promotes HSP90 Binding. J. Biol. Chem..

[54] Abravaya K., Myers M.P., Murphy S.P., Morimoto R.I. (1992). The Human Heat Shock Protein Hsp70 Interacts with HSF, the Transcription Factor That Regulates Heat Shock Gene Expression. Genes Dev..

[55] Krakowiak J., Zheng X., Patel N., Feder Z.A., Anandhakumar J., Valerius K., Gross D.S., Khalil A.S., Pincus D. (2018). Hsf1 and Hsp70 Constitute a Two-Component Feedback Loop That Regulates the Yeast Heat Shock Response. Elife.

[56] Kmiecik S.W., Le Breton L., Mayer M.P. (2020). Feedback Regulation of Heat Shock Factor 1 (Hsf1) Activity by Hsp70-mediated Trimer Unzipping and Dissociation from DNA. EMBO J..

[57] Masser A.E., Kang W., Roy J., Kaimal J.M., Quintana-Cordero J., Friedländer M.R., Andréasson C. (2019). Cytoplasmic Protein Misfolding Titrates Hsp70 to Activate Nuclear Hsf1. Elife.

[58] Roh S.H., Kasembeli M., Bakthavatsalam D., Chiu W., Tweardy D.J. (2015). Contribution of the Type II Chaperonin, TRiC/CCT, to Oncogenesis. Int. J. Mol. Sci..

[59] Neef D.W., Jaeger A.M., Gomez-Pastor R., Willmund F., Frydman J., Thiele D.J. (2014). A Direct Regulatory Interaction between Chaperonin TRiC and Stress-Responsive Transcription Factor HSF1. Cell Rep..

[60] Kumar S., Basu M., Ghosh M.K. (2022). Chaperone-Assisted E3 Ligase CHIP: A Double Agent in Cancer. Genes Dis..

[61] Dai Q., Zhang C., Wu Y., McDonough H., Whaley R.A., Godfrey V., Li H.H., Madamanchi N., Xu W., Neckers L. (2003). CHIP Activates HSF1 and Confers Protection against Apoptosis and Cellular Stress. EMBO J..

[62] Kim S.A., Yoon J.H., Kim D.K., Kim S.G., Ahn S.G. (2005). CHIP Interacts with Heat Shock Factor 1 during Heat Stress. FEBS Lett..

[63] Huang C.Y., Kuo W.W., Lo J.F., Ho T.J., Pai P.Y., Chiang S.F., Chen P.Y., Tsai F.J., Tsai C.H., Huang C.Y. (2016). Doxorubicin Attenuates CHIP-Guarded HSF1 Nuclear Translocation and Protein Stability to Trigger IGF-IIR-Dependent Cardiomyocyte Death. Cell Death Dis..

[64] Mariotto E., Viola G., Zanon C., Aveic S. (2020). A BAG’s Life: Every Connection Matters in Cancer. Pharmacol. Ther..

[65] Jin Y.H., Ahn S.G., Kim S.A. (2015). BAG3 Affects the Nucleocytoplasmic Shuttling of HSF1 upon Heat Stress. Biochem. Biophys. Res. Commun..

[66] Kizilboga T., Özden C., Can N.D., Onay Ucar E., Dinler Doganay G. (2024). Bag-1-Mediated HSF1 Phosphorylation Regulates Expression of Heat Shock Proteins in Breast Cancer Cells. FEBS Open Bio.

[67] Joutsen J., Sistonen L. (2019). Tailoring of Proteostasis Networks with Heat Shock Factors. Cold Spring Harb. Perspect. Biol..

[68] Hietakangas V., Ahlskog J.K., Jakobsson A.M., Hellesuo M., Sahlberg N.M., Holmberg C.I., Mikhailov A., Palvimo J.J., Pirkkala L., Sistonen L. (2003). Phosphorylation of Serine 303 Is a Prerequisite for the Stress-Inducible SUMO Modification of Heat Shock Factor 1. Mol. Cell. Biol..

[69] Westerheide S.D., Anckar J., Stevens S.M., Sistonen L., Morimoto R.I. (2009). Stress-Inducible Regulation of Heat Shock Factor 1 by the Deacetylase SIRT. Science.

[70] Tsai P.J., Lai Y.H., Manne R.K., Tsai Y.S., Sarbassov D., Lin H.K. (2022). Akt: A Key Transducer in Cancer. J. Biomed. Sci..

[71] Lu W.C., Omari R., Ray H., Wang J., Williams I., Jacobs C., Hockaden N., Bochman M.L., Carpenter R.L. (2022). AKT1 Mediates Multiple Phosphorylation Events That Functionally Promote HSF1 Activation. FEBS J..

[72] Carpenter R.L., Sirkisoon S., Zhu D., Rimkus T., Harrison A., Anderson A., Paw I., Qasem S., Xing F., Liu Y. (2017). Combined Inhibition of AKT and HSF1 Suppresses Breast Cancer Stem Cells and Tumor Growth. Oncotarget.

[73] Cigliano A., Wang C., Pilo M.G., Szydlowska M., Brozzetti S., Latte G., Pes G.M., Pascale R.M., Seddaiu M.A., Vidili G. (2017). Inhibition of HSF1 Suppresses the Growth of Hepatocarcinoma Cell Lines in Vitro and AKT-Driven Hepatocarcinogenesis in Mice. Oncotarget.

[74] Liu G.Y., Sabatini D.M. (2020). MTOR at the Nexus of Nutrition, Growth, Ageing and Disease. Nat. Rev. Mol. Cell Biol..

[75] Laplante M., Sabatini D.M. (2012). MTOR Signaling in Growth Control and Disease. Cell.

[76] Chou S.D., Prince T., Gong J., Calderwood S.K. (2012). mTOR Is Essential for the Proteotoxic Stress Response, HSF1 Activation and Heat Shock Protein Synthesis. PLoS ONE.

[77] Li J., Song P., Jiang T., Dai D., Wang H., Sun J., Zhu L., Xu W., Feng L., Shin V.Y. (2018). Heat Shock Factor 1 Epigenetically Stimulates Glutaminase-1-Dependent mTOR Activation to Promote Colorectal Carcinogenesis. Mol. Ther..

[78] Song Y., Bi Z., Liu Y., Qin F., Wei Y., Wei X. (2023). Targeting RAS–RAF–MEK–ERK Signaling Pathway in Human Cancer: Current Status in Clinical Trials. Genes Dis..

[79] Neuzillet C., Tijeras-Raballand A., De Mestier L., Cros J., Faivre S., Raymond E. (2014). MEK in Cancer and Cancer Therapy. Pharmacol. Ther..

[80] Tang Z., Dai S., He Y., Doty R.A., Shultz L.D., Sampson S.B., Dai C. (2015). MEK Guards Proteome Stability and Inhibits Tumor-Suppressive Amyloidogenesis via HSF1. Cell.

[81] Wales C.T.K., Taylor F.R., Higa A.T., McAllister H.A., Jacobs A.T. (2015). ERK-Dependent Phosphorylation of HSF1 Mediates Chemotherapeutic Resistance to Benzimidazole Carbamates in Colorectal Cancer Cells. Anticancer Drugs.

[82] Dai S., Tang Z., Cao J., Zhou W., Li H., Sampson S., Dai C. (2015). Suppression of the HSF 1-mediated Proteotoxic Stress Response by the Metabolic Stress Sensor AMPK. EMBO J..

[83] Su K.H., Dai S., Tang Z., Xu M., Dai C. (2019). Heat Shock Factor 1 Is a Direct Antagonist of AMP-Activated Protein Kinase. Mol. Cell.

[84] Chen K., Qian W., Li J., Jiang Z., Cheng L., Yan B., Cao J., Sun L., Zhou C., Lei M. (2017). Loss of AMPK Activation Promotes the Invasion and Metastasis of Pancreatic Cancer through an HSF1-Dependent Pathway. Mol. Oncol..

[85] Kovács D., Sigmond T., Hotzi B., Bohár B., Fazekas D., Deák V., Vellai T., Barna J. (2019). HSF1Base: A Comprehensive Database of HSF1 (Heat Shock Factor 1) Target Genes. Int. J. Mol. Sci..

[86] Xu M., Lin L., Ram B.M., Shriwas O., Chuang K.H., Dai S., Su K.H., Tang Z., Dai C. (2023). Heat Shock Factor 1 (HSF1) Specifically Potentiates c-MYC-Mediated Transcription Independently of the Canonical Heat Shock Response. Cell Rep..

[87] McMahon S.B., Van Buskirk H.A., Dugan K.A., Copeland T.D., Cole M.D. (1998). The Novel ATM-Related Protein TRRAP Is an Essential Cofactor for the c- Myc and E2F Oncoproteins. Cell.

[88] Fujimoto M., Takii R., Matsumoto M., Okada M., Nakayama K.I., Nakato R., Fujiki K., Shirahige K., Nakai A. (2022). HSF1 Phosphorylation Establishes an Active Chromatin State via the TRRAP–TIP60 Complex and Promotes Tumorigenesis. Nat. Commun..

[89] Fujimoto M., Takii R., Takaki E., Katiyar A., Nakato R., Shirahige K., Nakai A. (2017). The HSF1-PARP13-PARP1 Complex Facilitates DNA Repair and Promotes Mammary Tumorigenesis. Nat. Commun..

[90] Fujimoto M., Takii R., Katiyar A., Srivastava P., Nakai A. (2018). Poly(ADP-Ribose) Polymerase 1 Promotes the Human Heat Shock Response by Facilitating Heat Shock Transcription Factor 1 Binding to DNA. Mol. Cell. Biol..

[91] Huang M., Dong W., Xie R., Wu J., Su Q., Li W., Yao K., Chen Y., Zhou Q., Zhang Q. (2022). HSF1 Facilitates the Multistep Process of Lymphatic Metastasis in Bladder Cancer via a Novel PRMT5-WDR5-Dependent Transcriptional Program. Cancer Commun..

[92] Björk J.K., Åkerfelt M., Joutsen J., Puustinen M.C., Cheng F., Sistonen L., Nees M. (2016). Heat-Shock Factor 2 Is a Suppressor of Prostate Cancer Invasion. Oncogene.

[93] Smith R.S., Takagishi S.R., Amici D.R., Metz K., Gayatri S., Alasady M.J., Wu Y., Brockway S., Taiberg S.L., Khalatyan N. (2022). HSF2 Cooperates with HSF1 to Drive a Transcriptional Program Critical for the Malignant State. Sci. Adv..

[94] Kawamura G., Hattori M., Takamatsu K., Tsukada T., Ninomiya Y., Benjamin I., Sassone-Corsi P., Ozawa T., Tamaru T. (2018). Cooperative Interaction among BMAL1, HSF1, and P53 Protects Mammalian Cells from UV Stress. Commun. Biol..

[95] Blagosklonny M.V., Toretsky J., Bohen S., Neckers L. (1996). Mutant Conformation of P53 Translated in Vitro or in Vivo Requires Functional HSP90. Proc. Natl. Acad. Sci. USA.

[96] Walerych D., Olszewski M.B., Gutkowska M., Helwak A., Zylicz M., Zylicz A. (2009). Hsp70 Molecular Chaperones Are Required to Support P53 Tumor Suppressor Activity under Stress Conditions. Oncogene.

[97] King F.W., Wawrzynow A., Höhfeld J., Zylicz M. (2001). Co-Chaperones Bag-1, Hop and Hsp40 Regulate Hsc70 and Hsp90 Interactions with Wild-Type or Mutant P53. EMBO J..

[98] Wiech M., Olszewski M.B., Tracz-Gaszewska Z., Wawrzynow B., Zylicz M., Zylicz A. (2012). Molecular Mechanism of Mutant P53 Stabilization: The Role of HSP70 and MDM2. PLoS ONE.

[99] Isermann T., Şener Ö.Ç., Stender A., Klemke L., Winkler N., Neesse A., Li J., Wegwitz F., Moll U.M., Schulz-Heddergott R. (2021). Suppression of HSF1 Activity by Wildtype P53 Creates a Driving Force for P53 Loss-of-Heterozygosity. Nat. Commun..

[100] Li D., Yallowitz A., Ozog L., Marchenko N. (2014). A Gain-of-Function Mutant P53-HSF1 Feed Forward Circuit Governs Adaptation of Cancer Cells to Proteotoxic Stress. Cell Death Dis..

[101] Nguyen C.H., Lang B.J., Chai R.C.C., Vieusseux J.L., Kouspou M.M., Price J.T. (2013). Heat-Shock Factor 1 Both Positively and Negatively Affects Cellular Clonogenic Growth Depending on P53 Status. Biochem. J..

[102] Nadeau K., Das A., Walsh C.T. (1993). Hsp90 Chaperonins Possess ATPase Activity and Bind Heat Shock Transcription Factors and Peptidyl Prolyl Isomerases. J. Biol. Chem..

[103] Pasqua A.E., Sharp S.Y., Chessum N.E.A., Hayes A., Pellegrino L., Tucker M.J., Miah A., Wilding B., Evans L.E., Rye C.S. (2023). HSF1 Pathway Inhibitor Clinical Candidate (CCT361814/NXP800) Developed from a Phenotypic Screen as a Potential Treatment for Refractory Ovarian Cancer and Other Malignancies. J. Med. Chem..

[104] Li X., Srinivasan S.R., Connarn J., Ahmad A., Young Z.T., Kabza A.M., Zuiderweg E.R.P., Sun D., Gestwicki J.E. (2013). Analogues of the Allosteric Heat Shock Protein 70 (Hsp70) Inhibitor, MKT-077, as Anti-Cancer Agents. ACS Med. Chem. Lett..

[105] Sverchinsky D.V., Alhasan B.A., Mikeladze M.A., Lazarev V.F., Kuznetcova L.S., Morshneva A.V., Nikotina A.D., Ziewanah A., Koludarova L.V., Starkova T.Y. (2023). Autocrine Regulation of Tumor Cell Repopulation by Hsp70-HMGB1 Alarmin Complex. J. Exp. Clin. Cancer Res..

[106] Li Z.N., Luo Y. (2023). HSP90 Inhibitors and Cancer: Prospects for Use in Targeted Therapies (Review). Oncol. Rep..

[107] Chen Y., Chen J., Loo A., Jaeger S., Bagdasarian L., Yu J., Chung F., Korn J., Ruddy D., Guo R. (2013). Targeting HSF1 Sensitizes Cancer Cells to HSP90 Inhibition. Oncotarget.

[108] Kudryavtsev V.A., Khokhlova A.V., Mosina V.A., Selivanova E.I., Kabakov A.E. (2017). Induction of Hsp70 in Tumor Cells Treated with Inhibitors of the Hsp90 Activity: A Predictive Marker and Promising Target for Radiosensitization. PLoS ONE.

[109] Do K., Speranza G., Chang L.C., Polley E.C., Bishop R., Zhu W., Trepel J.B., Lee S., Lee M.J., Kinders R.J. (2015). Phase I Study of the Heat Shock Protein 90 (Hsp90) Inhibitor Onalespib (AT13387) Administered on a Daily for 2 Consecutive Days per Week Dosing Schedule in Patients with Advanced Solid Tumors. Investig. New Drugs.

[110] Pesonen L., Svartsjö S., Bäck V., de Thonel A., Mezger V., Sabéran-Djoneidi D., Roos-Mattjus P. (2021). Gambogic Acid and Gambogenic Acid Induce a Thiol-Dependent Heat Shock Response and Disrupt the Interaction between HSP90 and HSF1 or HSF2. Cell Stress Chaperones.

[111] Rastogi S., Joshi A., Sato N., Lee S., Lee M.J., Trepel J.B., Neckers L. (2024). An Update on the Status of HSP90 Inhibitors in Cancer Clinical Trials. Cell Stress Chaperones.

[112] Wang M., Zou J., Wang J., Liu M., Liu K., Wang N., Wang K. (2022). Aberrant HSF1 Signaling Activation Underlies Metformin Amelioration of Myocardial Infarction in Mice. Mol. Ther. Nucleic Acids.

[113] Wong S.H.D., Yin B., Li Z., Yuan W., Zhang Q., Xie X., Tan Y., Wong N., Zhang K., Bian L. (2023). Mechanical Manipulation of Cancer Cell Tumorigenicity via Heat Shock Protein Signaling. Sci. Adv..

[114] Timofeev O., Giron P., Lawo S., Pichler M., Noeparast M. (2024). ERK Pathway Agonism for Cancer Therapy: Evidence, Insights, and a Target Discovery Framework. NPJ Precis. Oncol..

[115] Satoh R., Hagihara K., Matsuura K., Manse Y., Kita A., Kunoh T., Masuko T., Moriyama M., Moriyama H., Tanabe G. (2017). Identification of ACA-28, a 1′-Acetoxychavicol Acetate Analogue Compound, as a Novel Modulator of ERK MAPK Signaling, Which Preferentially Kills Human Melanoma Cells. Genes Cells.

